# Investigation of the Initial Host Response of Naïve Atlantic Salmon (*Salmo salar*) Inoculated with *Paramoeba perurans*

**DOI:** 10.3390/microorganisms9040746

**Published:** 2021-04-02

**Authors:** Michelle McCormack, Eugene Dillon, Ian O’Connor, Eugene MacCarthy

**Affiliations:** 1 Marine and Freshwater Research Centre, Galway Mayo Institute of Technology, Dublin Road, H91 TRNW Galway, Ireland; Ian.OConnor@gmit.ie (I.O.); Eugene.McCarthy@gmit.ie (E.M.); 2 Conway Institute of Biomolecular & Biomedical Research, University College Dublin, D04 V1W8 Dublin, Ireland; eugene.dillon@ucd.ie

**Keywords:** amoebic gill disease, Atlantic salmon, gel-free proteomics, gill proteome, serum proteome

## Abstract

Amoebic Gill Disease (AGD), caused by the ectoparasite *Paramoeba perurans* is characterised by hyperplasia of the gill epithelium and lamellar fusion. In this study, the initial host response of naïve Atlantic salmon (*Salmo salar*) inoculated with *P. perurans* was investigated. Using gel-free proteomic techniques and mass spectrometry gill and serum samples were analysed at 7 timepoints (2, 3, 4, 7, 9, 11 and 14 days) post-inoculation with *P. perurans*. Differential expression of immune related proteins was assessed by comparison of protein expression from each time point against naïve controls. Few host immune molecules associated with innate immunity showed increased expression in response to gill colonisation by amoebae. Furthermore, many proteins with roles in immune signalling, phagocytosis and T-cell proliferation were found to be inhibited upon disease progression. Initially, various immune factors demonstrated the anticipated increase in expression in response to infection in the serum while some immune inhibition became apparent at the later stages of disease progression. Taken together, the pro-immune trend observed in serum, the lack of a robust early immune response in the gill and the diversity of those proteins in the gill whose altered expression negatively impact the immune response, support the concept of a pathogen-derived suppression of the host response.

## 1. Introduction

Amoebic Gill Disease (AGD), first described in Tasmania in 1986 [1], is caused by the marine ectoparasite *Paramoeba perurans*, and as the number of cases reported worldwide has escalated in recent years, it has become recognised as one of the most significant health threats in salmon farming [2]. Diseased fish exhibit multifocal gill hyperplasia, lamellar fusion, increased mucus production and macroscopic lesions on gill filaments [3]. The resultant respiratory distress can lead to mortality in up to 80% of cases if left untreated [4]. Treatments are limited to fresh water or hydrogen peroxide baths, which effectively remove the amoebae from affected gills and reduce gill mucus viscosity [5,6], however neither treatment prevents re-infection [7]. The economic impact of AGD is not insignificant, with an estimated 20% additional production costs being placed on farmers as a direct consequence of fish loss and treatment [8].

Several studies have focused on gill gene expression analyses in order to investigate host response in AGD. Some have highlighted a transcriptional down-regulation of key immune factors, such as interferon induced MHC I and MHC II pathways and anti-oxidant enzymes [9,10], while others have noted up-regulation of pro-inflammatory cytokine and immune-regulatory gene transcription [11,12] as a typical response to AGD. Some of the most significant genomic responses to the disease were found to be lesion specific [12,13], which, when compared with data obtained from non-lesion areas, appear contradictory. This has led to the contention that genetic screening focusing on lesion tissue, without adequately accounting for infiltration of immune factors from surrounding cells, could give rise to results, which may, if interpreted in the context of the entire gill, misrepresent the cellular response to the parasitic infection [14]. However, it has also been demonstrated that the cellular composition and ratio of cell types within the gill are subject to variation dependent on disease status, developmental stage and experimental conditions, and these may also account for some of the variations observed between studies [15].

Other studies have reported on serum immune parameters in AGD infected salmon, though many of the traditional factors such as immunoglobulins and immune related enzymes remained unaltered or yielded varying results in the disease condition [16,17,18]. Although the work done to date has made progress towards understanding AGD, differences in disease stage, amoebic load, exposure duration and environmental conditions make comparisons between studies difficult and further work is required to fully elucidate the host response in AGD from early to late disease stage, at both a local and systemic level.

In aquaculture, proteomics has gained recognition as a technique which can yield valuable information regarding a biological system and provide greater understanding of the pathophysiology of disease, than genomics alone [19]. Previously, proteomic analysis of salmon gill and skin mucus implicated several inflammatory pathways and immune molecules in the host response to repeated *P. perurans* infection [20]. Marcos-Lopez et al. [18] were the first to use gel-based proteomics to investigate disease progression in salmon gills across 6 sequential timepoints in AGD affected fish and highlighted proteins involved in cell proliferation, immunity, oxidative metabolism and cytoskeletal reorganisation as being among those most significantly altered in the disease state.

Limitations of the traditional 2DPAGE proteomics, particularly with respect to gel to gel variation and limited proteome coverage [19,21], have led to the rise in popularity of gel free proteomic techniques [22,23]. Therefore, in this study whole gill arches and blood samples from salmon with AGD, from 7 time points spanning sub-clinical and clinical stages of the disease, were submitted to a filter-based sample preparation and in-solution tryptic digestion followed by label-free mass spectrometry analysis [22,24]. Analysis of differential gill and serum protein expression across all time points, in comparison to control fish will contribute to the understanding of the pathophysiology of this disease, and the salmon host response to the infection at both a local and systemic level.

## 2. Materials and Methods

### 2.1. Fish Husbandry

Atlantic salmon reared on a land-based freshwater hatchery in the west of Ireland, were transferred to a land-based indoor, marine recirculating facility at the Marine and Freshwater Research Centre (MFRC) at the Galway-Mayo Institute of Technology (GMIT) in Galway, Ireland following smoltification. Salmon smolts (average weight 70 g) were distributed into four circular black 1000 L tanks (*n* = 45/tank) with the following conditions: stocking density 3.6 kg m^−3^, water temperature 12 °C, artificial salinity of 30 parts per thousand (ppt) (Coral Pro salt, Red Sea), light cycle of 12 h light, 12 h dark. Fish were fed a commercial salmon diet at 1% body weight per day. This project was authorised by the Health Products Regulatory Authority (HPRA) in Ireland under project authorisation number AE19137/P001, in compliance with the requirements under Directive 2010/63/EU transposed into Irish law by the European Union (Protection of Animals Used for Scientific Purposes) Regulations 2012 (S.I. No 543 of 2012 as amended).

### 2.2. Paramoeba perurans Challenge and Sample Collection

Following an acclimatisation period of 6 days, 90 fish (45 × 2) were challenged with *P. perurans* (2750 amoebae/L) in a volume of 300 L for 4 h with oxygen saturation. This challenge load was similar to that employed in other studies which successfully induced AGD [12,25]. Fish behaviour and welfare were closely monitored. Control fish, also 90 fish (45 × 2), were also held in 300 L for 4 h. Following the challenge period, the control and treatment fish were moved into new tanks containing 1000 L seawater. To confirm the identity of the amoebae, real-time polymerase chain reaction (PCR) was carried out, as described by Downes [26], at the National Reference Laboratory for finfish disease, Marine Institute, Galway, Ireland.

Gill and blood samples were collected from four control salmon pre-challenge (0 days post infection (dpi)) and then a further four at each of the following timepoints post *P. perurans* challenge: 2 dpi, 3 dpi, 4 dpi, 7 dpi, 9 dpi, 11 dpi and 14 dpi. Fish were euthanised by overdose of anaesthetic (400 mg/L tricaine methanesulfonate (MS-222)) and gills scored in accordance with the standard commercial protocol, adapted from Taylor et al. [3]. For histological analyses, the first gill arch on the left side of each fish was excised and immediately fixed in 10% neutral buffered formalin. For gill proteomic analysis, the 2nd right gill arch from each fish was excised, the arch cartilage removed, and the remaining gill filaments snap frozen in dry ice and kept at −80 °C until required. For serum proteomic analysis, blood was obtained from the caudal vein of each specimen and incubated at 4 °C overnight. Blood samples were spun at 10,000× *g* for 5 min at 4 °C and the serum was retained and stored at −80 °C until required.

### 2.3. Histology

Fixed gill samples were routinely processed and embedded in paraffin wax blocks. Sections (5 μm) were stained with haematoxylin and eosin (H&E) and examined using an Olympus BX41 Microscope and CellSens software (Olympus, Tokyo, Japan). Gill filaments exhibiting features of AGD including lamellar fusion, hyperplasia and vesicle formation were imaged.

### 2.4. Proteomics FASP

Gills were weighed, and tissue lysis was performed, on ice, by sonication (4 × 30 s cycles at 25% amplitude; Fisherbrand™ Q500 Sonicator, Waltham, MA, USA), in a 50 mM Tris, 1% SDS lysis buffer. Protein quantification was carried out using the Pierce BCA assay (Thermo Scientific) and 300 µg of protein were incubated with 0.1 M DTT at 95 °C for 10 min. Serum samples were thawed, and protein quantification was carried out using the Pierce BCA assay. In total, 300 µg of protein were diluted 1 in 10 in a 50 mM Tris, 1% SDS lysis buffer and incubated with 0.1 M DTT at 95 °C for 10 min. For both gill and serum samples, clean up and digestion steps were carried out using the FASP method based on that described by Wiśniewski et al. [24]. Briefly, samples were mixed with UA buffer (8 M urea, 100 mM Tris—HCl pH 8.9) and contaminants and salts were removed by repeated ultrafiltration through 10 kDa Vivacon centrifugal concentrators (Sartorius, Göttingen, Germany). Samples were incubated for 20 min in darkness with 100 µL 50 mM iodoacetamide in UA buffer, to facilitate blocking of reduced cysteine residues. After further washes with UA and 50 mM ammonium bicarbonate (ABC), trypsin (1 µg per 100 µg protein; Sigma-Aldrich, St. Louis, MO, USA) in 40 µL ABC was added to the samples in the concentrator units. Digestion was performed at 37 °C for 4 h. The resulting peptide fragments were washed with ABC and digestion was halted by the addition of 5 µL 50% acetic acid. The eluates were desalted on C18—Stage tips (Sigma-Aldrich) [27], dried in a vacuum concentrator (Eppendorf Vacufuge concentrator 5301) and stored at −20 °C until mass spectrometry analysis.

### 2.5. Mass Spectrometry

The samples were analysed by the Mass Spectrometry Resource (MSR) in University College, Dublin on a Thermo Scientific Q Exactive mass spectrometer connected to a Dionex Ultimate 3000 (RSLCnano, Waltham, MA, USA) chromatography system. Peptides were separated on C18 home-made column (C18RP Reprosil-Pur, Ammerbuch, Germany, 100 × 0.075 mm × 3 μm) over 60 min at a flow rate of 250 nL/min with a linear gradient of increasing acetonitrile (ACN) from 1% to 27%. The mass spectrometer was operated in data-dependent mode; a high resolution (70,000) MS scan (300–1600 *m*/*z*) was performed to select the twelve most intense ions and fragmented using high energy C-trap dissociation for MS/MS analysis.

### 2.6. Data Processing and Bioinformatics

Raw data from the Q-Exactive were processed using the MaxQuant [28,29] (version 1.6.4.0) incorporating the Andromeda search engine [30]. To identify peptides and proteins, MS/MS spectra were matched against the Uniprot *Salmo salar* database (2019_04) containing 82,390 entries. All searches were performed using the default setting of MaxQuant, with trypsin as specified enzyme allowing two missed cleavages and a false discovery rate of 1% on the peptide and protein level. The database searches were performed with carbamidomethyl (C) as fixed modification and acetylation (protein N terminus) and oxidation (M) as variable modifications. For the generation of label free quantitative (LFQ) ion intensities for protein profiles, signals of corresponding peptides in different nano-HPLC MS/MS runs were matched by MaxQuant in a maximum time window of 1 min [31]. The mass spectrometry proteomics data have been deposited to the ProteomeXchange Consortium via the PRIDE [32] partner repository with the dataset identifier PXD022101.

### 2.7. Data Analysis

Data were processed using the Perseus (v 1.6.7.0) data analysis suite (https://maxquant.net/perseus/ (accessed on 9 January 2020)). Label free quantitative (LFQ) ion current intensities were transformed (log_2_). Imputation was carried out to replace missing values with numbers drawn from a normal distribution. A Student’s *t*-test was performed to determine statistical significance (*p* < 0.05). For visualisation using heatmaps, data were z-score normalised. Fold change values were obtained by calculating the difference between the two compared conditions of the mean log_2_ transformed values, and the Student’s *t*-test *p*-value. Pathway enrichment analysis was performed using the ClueGo (v 2.5.4) [33] and Cluepedia (v 1.5.7) [34] plugins in Cytoscape (v 3.7.1) [35] with the *salmo salar* (8030) marker set. The gene ontology immune system process, consisting of 466 genes, was used [36,37]. GO tree levels (min = 3; max = 8) and GO term restriction (min#genes = 3, min% = 4%) were set and terms were grouped using a Kappa Score Threshold of 0.4. The classification was performed by the two-side hypergeometric statistic test, and the probability value was corrected by the Bonferroni method (adjusted % term *p*-value < 0.05) [33].

## 3. Results

### 3.1. In Vivo Challenge, RT-PCR and Histopathology

**Figure 1:**
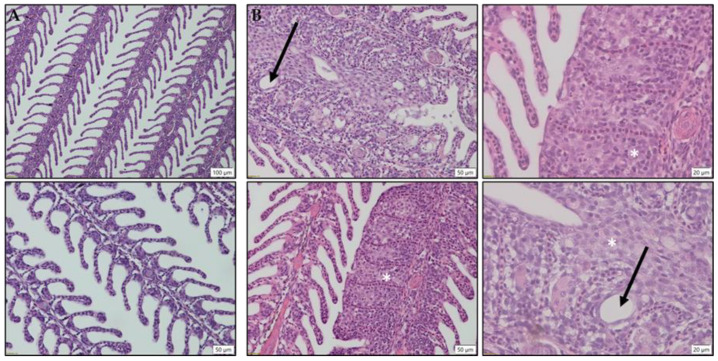
Histology (H&E staining) of salmon gill samples: (**A**) Gill samples from pre-inoculation controls and (**B**) gills from salmon sampled 14 dpi. Features commonly associated with AGD including Hyperplastic lesions, hyperplastic lamellar fusion (*) and interlamellar vesicles (black arrows) and were evident in gill samples at 14 dpi. Scale bars are indicated on images.

The identity of the amoebae was confirmed by real-time polymerase chain reaction (PCR) as described by Downes et al. [26]. By 2 dpi, 5 of 6 fish tested positive for the presence of *P. perurans*. At 7 dpi, all 6 fish tested were positive for *P. perurans*. The first lesions were observed at 11 dpi. Gill scoring was undertaken in accordance with the standard commercial protocol adapted from Taylor et al. [3], and, in compliance with regulations, the trial was halted when a gill score of 2 was achieved by 50% of fish. Histopathological analysis revealed hyperplastic lesions, interlamellar vesicles and hyperplastic lamellar fusion at 14 dpi (Figure 1).

### 3.2. Gill Proteomics

**Figure 2:**
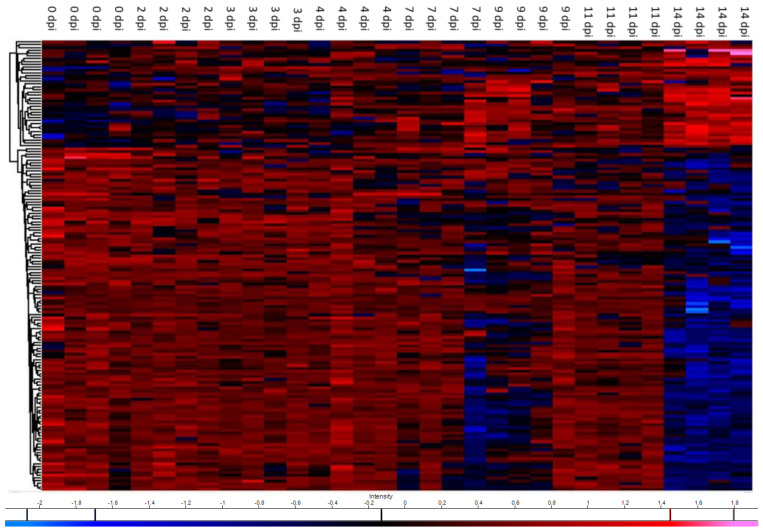
Heatmap plot of the proteins exhibiting *t*-test differences of at least +/− 1.5 in control and post infection samples after LFQ values were z-score normalised. Hierarchical clustering was performed using Euclidian distance and average linkage using the Perseus software.

**Figure 3:**
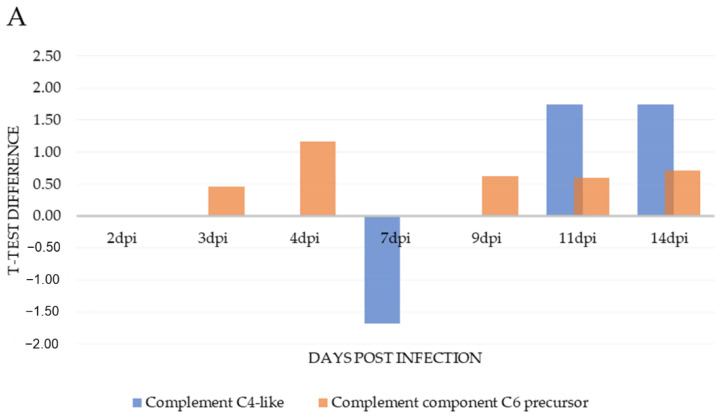

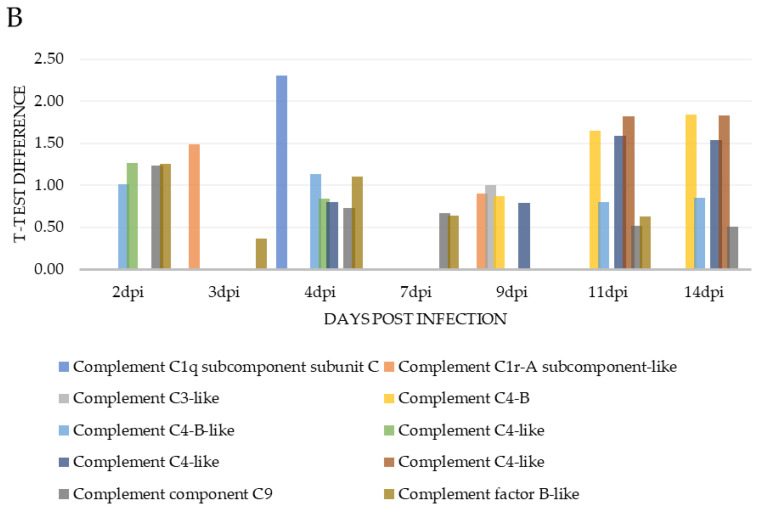
Pro-immune complement factors: *t*-test difference values for pro-immune complement factors in gill (**A**) and serum (**B**) samples across 7 timepoints relative to control values.

**Figure 4:**
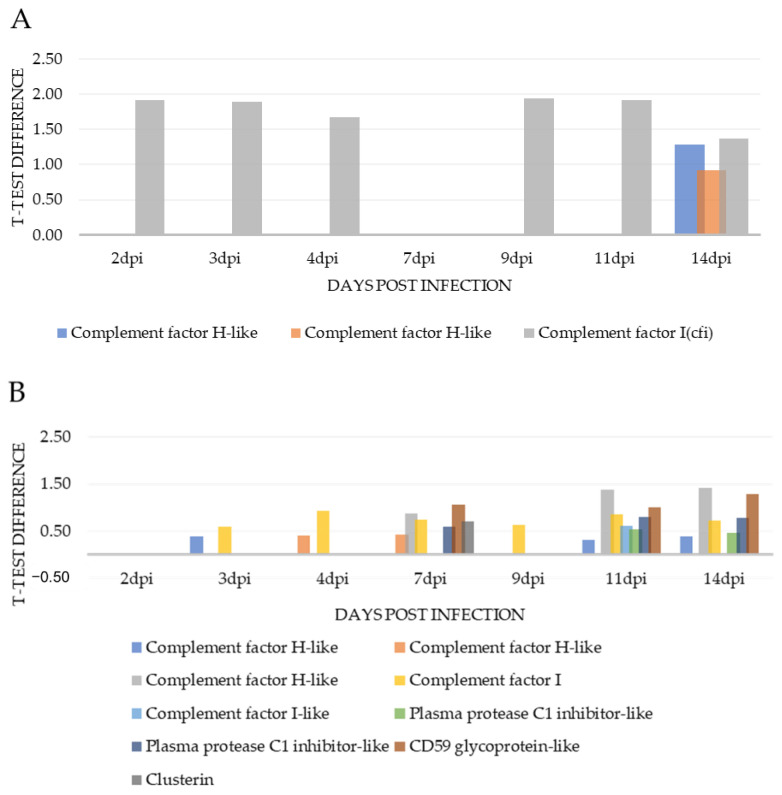
Immuno-inhibitory complement factors: *t*-test difference values for complement inhibitory factors in gill (**A**) and serum (**B**) samples across 7 timepoints relative to control values.

In excess of 5000 proteins were initially identified by mass spectrometry. Upon filtering based on matching more than one peptide in each case and performing Student’s *t*-test statistics on normalised and imputed data, 180 proteins were found to be differentially expressed at 2 dpi, 178 proteins at 3 dpi, 136 proteins at 4 dpi, 239 proteins at 7 dpi, 212 proteins at 9 dpi, 335 proteins at 11 dpi and 667 proteins at 14 dpi (*p* < 0.05). The number of proteins differentially expressed was highest at 14 dpi, which is in line with the progression of classic disease features, as confirmed by gill scoring and histopathology analyses. Between 13% and 29% of these proteins exhibited a *t*-test difference of at least +/− 1.5, across the 7 time points, a total of 393 proteins (Figure 2). In the gill, less than 10% of differentially expressed proteins were components of the host immune system. *t*-test difference values across all timepoints for proteins involved in immunity are displayed in Table 1. The differential expression of those gill proteins which have a role in complement stimulation and complement inhibition, across all time points, relative to control values, are displayed in Figure 3A and Figure 4A, respectively. Immune system process pathway analysis of gill samples, carried out using the Cluego app (v 2.5.7) in Cytoscape (v 1.5.7), identified no immune pathway enrichment at any timepoint in gill samples.

### 3.3. Serum Proteomics

In excess of 600 proteins were initially identified by mass spectrometry. Upon filtering based on matching more than one peptide in each case and performing Students’ *t*-test statistics on normalised and imputed data, 28 proteins were found to be differentially expressed at 2 dpi, 25 proteins at 3 dpi, 41 proteins at 4 dpi, 31 proteins at 7 dpi, 40 proteins at 9 dpi, 70 proteins at 11 dpi and 81 proteins at 14 dpi (*p* < 0.05). As in the gill, the number of proteins differentially expressed was highest at 14 dpi. Between 24% and 75% of these proteins exhibited a *t*-test difference of at least +/− 1.2, across the 7 time points, a total of 119 proteins. Approximately 47% of differentially expressed serum proteins had immune related functions. Of these, 64% were involved in the complement cascade, or were members of the apolipoprotein family. *t*-test difference values across all timepoints for those proteins involved in immunity are detailed in Table 2. The differential expression of those serum proteins which have a role in complement stimulation and complement inhibition, across all time points, relative to control values, are displayed in Figure 3B and Figure 4B, respectively. Immune system process pathway analysis of serum samples was carried out using the Cluego app (v 2.5.7) in Cytoscape (v 1.5.7). Up-regulation of pro-immune pathways was evident at 3, 4 and 7 dpi (Supplemental Figure S1A–C). Immune enrichment was absent at 9 dpi, but was restored by 11 dpi (Supplemental Figure S1D). At 11 dpi and 14 dpi, the pro-immune pathway up-regulation was accompanied by an elevation of various pathways which negatively regulate immunity (Supplemental Figure S1D,E).

## 4. Discussion

Immune response is an intricate and dynamic phenomenon, which can be stimulated or inhibited by numerous factors often in a context-dependent manner. As a result, the investigation of the host response to AGD is a complex and multi-faceted challenge. Previous studies have taken alternate approaches to the investigation of AGD, with some focusing specifically on lesioned tissue, while others have looked at the entire gill area. In some cases genomic responses were found to be lesion specific, or more pronounced in the lesion [12], while others suggest that examination of the responses only in lesions may fail to account for infiltration of immune factors from surrounding tissue [14]. This study investigated the host immune response across various timepoints from very early sub-clinical disease stages until such point as gill score 2 was achieved in 50% of fish, in compliance with the HPRA experimental licence. Given that our earliest sampled timepoint was 2 dpi and lesions did not develop until 11 dpi, it was therefore essential to utilise a consistent sampling approach to investigate the host proteome response in the gill tissue. Indeed, although lesion specific reactions may not be evident in our study, in the context of investigating the host response from sub-clinical disease stages, the delay between initial inoculation and eventual development of visible lesions necessitates the inclusion of the entire gill. We therefore had the opportunity to gain insight into early stage changes at a protein level across the entire gill and serum.

Innate immunity in fish is the primary defence system, central to their survival from early developmental stages. The complement system is particularly diverse in fish and elements of complement have been shown to be expressed from the early embryonic stage [38,39]. The range of complement factors observed to be differentially expressed in serum across various timepoints in this study reflects this diversity. Serum pro-immune complement activity included many factors from both the classic and alternative pathways showing some degree of up-regulation. However, in the gill, immuno-stimulatory complement was limited to the expression of the C4 component and C6 precursor of the complement cascade. C4 was initially found to be significantly down-regulated at 7 dpi and only eventually up-regulated from 11 dpi and the expression of complement component C6 precursorwas only marginally increased across various timepoints.

Complement factor I (CFI) negatively regulates the complement system by cleavage of the C3b and C4b complement factors, both of which have been implicated in opsonization, stimulation of antibody response and clearance of immune complexes and apoptotic cells from tissues [40]. No clinical pathologies are associated with up-regulation of CFI and complement factor inactivation by CFI is strongly dependent on appropriate cofactor binding [41]. Therefore, the impact of CFI up-regulation must be investigated in the context of cofactor protein expression. However, the up-regulation of CFI expression, though observed in both tissue types across various timepoints in this study, was much more pronounced in the gill. Complement factor H (CFH), also a negative regulator of the complement cascade which binds to complement C3b blocking the downstream alternative pathway, may be particularly interesting in the context of AGD. In human pathogenic microbe studies pathogen expression of host-like glycans facilitates pathogen binding to complement factor H, which in turn stimulates host immune suppression as a strategy for microbe survival [42,43]. Various isoforms of CFH exhibit up-regulation in both tissue types, particularly towards the later stages in this study though expression in the gill is more elevated. The latest timepoint in this study is relatively early in terms of AGD disease progression. Therefore, investigation of the expression of CFI and CFH in fish at later stages of disease could prove interesting in the context of pathogen-derived suppression of the local host immune response.

Properdin stimulates the complement cascade, while clusterin, CD59 and the plasma protease C1 inhibitor inhibit complement [44,45,46]. In serum, while the expression of properdin, clusterin and both plasma protease inhibitor isoforms showed modest up-regulation, CD59 demonstrated a more substantial up-regulation which may point to enhanced cascade inhibition. However, the expressions of these proteins in the serum must be considered in the context of their roles in normal complement regulation and their interactions with the various other complement factors expressed in serum.

Taken together these data suggest that though modest in some cases, a variety of pro-immune factors are up-regulated in response to AGD and suggest an immune response at the systemic level. However, the dearth of complement factor expression, and the inhibitory action of those most substantially up-regulated suggest a local suppression of the complement cascade within gill tissue. It is noteworthy however, that previous studies have either failed to detect differences in serum complement factor activity [47], or have been unable to detect any discernible effects of variable complement expression in response to *P. perurans* colonisation [48]. Therefore, the significance of the complement cascade in the host response to AGD remains unclear. However, a recent genome mapping study implicated the C4 gene as one of three genes involved in disease resistance in AGD [49], and genetic screening of susceptible versus resistant fish also demonstrated an upregulation of plasma protease C1 inhibitor in resistant gill samples [50]. Kube et al. also demonstrated that resistance after one *P. perurans* infection was attributable to an innate response, in contrast to resistance after multiple infections [8]. In the context of these previous findings, our data may support the role of complement in the development of disease resistance in AGD.

Apolipoproteins, though traditionally associated with lipoprotein structure and metabolism, are emerging as significant components in innate immunity. The biological significance of apolipoprotein subtypes is varied and context-dependent. Apolipoprotein A-I (ApoA-I), one of the most abundant apolipoproteins in fish [51], and Apolipoprotein E (ApoE) have both been implicated in the binding and neutralization of bacterial lipopolysaccharide (LPS) in mammalian lung. Furthermore, ApoE can facilitate bacterial endocytosis and ApoA-I can stimulate Toll Like Receptor (TLR) pathways to promote further immune responses [52]. Apolipoprotein C-I (ApoC-I) facilitates the LPS induced inflammatory response in mouse models [53] and is down-regulated in severe sepsis [54]. Conversely, apolipoprotein A-IV (ApoA-IV) has an inhibitory effect on integrin regulated platelet adhesion [55], which can in turn negatively impact on leukocyte recruitment to the site of infection or injury [56]. Furthermore ApoA-IV has a negative impact on eosinophil induced inflammation and ApoA-I acts as a negative acute phase protein, which interferes with T-cell signalling and reduces inflammation. These proteins are considered sufficiently potent such that their use as therapeutic agents to reduce hyper responsiveness in airway allergies is being investigated [57]. Interpretation of apolipoprotein expression though inherently complex due to the diversity of roles, is further complicated by the finding that expression in response to bacterial infection in channel catfish was found to be highly pathogen-dependent [58].

In both the gill and serum, with the exception of the increase in expression of ApoA-I and ApoEb from 7 dpi, the differential expression values obtained for the other apolipoprotein isoforms may be indicative of an elevated inhibition of leukocyte recruitment and a reduction of the inflammatory response. Up-regulation of the pro-inflammatory cytokines tumour necrosis factor α (TNFα) and Interleukin 6 (IL-6) have previously been shown to decrease ApoA-IV expression at both the gene and protein level in obesity models in mice [59]. While upregulation of TNFα gene expression was previously demonstrated particularly in lesions [14], another study found TNFα isoforms to be unchanged or down-regulated in AGD-associated gill lesions at the gene level [60]. This is consistent with the lack of significant expression of pro-inflammatory cytokines detected at the protein level in either gill or serum in this study. Here, the elevated expression of ApoA-IV, evident in both gill and serum may be due to the loss of the negative regulation afforded by the pro-inflammatory cytokines. Other studies have found differential protein expression of some apolipoproteins [18,20], however this is the first study to report such a diverse range of apolipoprotein isoforms demonstrating differential expression in AGD. Indeed, the consistencies between gill and serum fold change trends observed across various timepoints in this study suggest the importance of these proteins in the host response, however the nature of the roles of apolipoproteins requires further investigation.

Within the gill, many other factors involved in host immune response, inflammation, and immune signalling were found to be differentially expressed. Histone H2A which acts as an antimicrobial peptide (AMP) [61] showed no significant activity until 14 dpi, at which point it was markedly down-regulated. Eosinophil Peroxidase (EPX) which can oxidise halide ions to form bactericidal reactive oxygen species [62,63] was downregulated from 4 dpi, which aside from potentially impacting on bactericidal ability, may also impact negatively on macrophage phagocytosis [64]. The marked down-regulation of leukocyte elastase inhibitor which itself impedes the action of proteolytic enzymes and inflammatory caspases as a mechanism of protection against aberrant protein degradation [65] may negatively impact on neutrophil survival, as previously demonstrated in mouse models [66].

Granulins which activate the TLR 9 [67], the leucine-rich repeat-containing protein 59 (LRRC 59) which has a role in TLR trafficking from the ER [68] and 1-phosphatidylinositol 4,5-bisphosphate phosphodiesterase delta-1 which catalyses IP3 and DAG production [69] all exhibited decreased expression in this study. The expressions of heterogeneous nuclear ribonucleoprotein A0 and Nuclear Receptor Coactivator 5, both with roles in the binding and endocytosis of bacterial DNA [70] and Ras-related protein Rab-11A, the down-regulation of which has previously been implicated in the vascular leakage observed in pulmonary microvessels during microbial sepsis [71], were also found to be reduced. Dicarbonyl and L-xylulose reductase catalyses the reduction of Reactive Carbonyl Species (RCS). Down-regulation of this enzyme as observed in our current study from 7 dpi results in dicarbonyl stress which can impact protein folding and mitochondrial dysfunction and has been identified as both a promoter and end product of oxidative stress [72]. In a recent human diabetes study, markers of dicarbonyl stress were associated with complement modulation though the biological impact was found to be stress marker specific [73]. Furthermore Magnesium transporter protein 1 (MGT1) is essential for magnesium homeostasis in eurkaryotic cells and Mg^2+^ has been reported to act as a second messenger in immunity [74]. A deficiency of MGT1, as observed here from 7 dpi, alters immune glycoprotein levels [75] and has been linked to impaired expression of programmed cell death and natural killer activating receptors in T-cells [76]. The functional diversity of these proteins and their downregulation demonstrates the range of immune functions negatively affected by *P. perurans* colonisation.

The lacklustre early immune response in the gill was somewhat counteracted by the discussed elevation of complement factor 4 at 11 dpi, ApoA-I at 7 dpi and the cation-dependent mannose—6-phosphate receptor which has a role in trafficking of lysosomal enzymes to the lysosome [77] at 4 dpi. Furthermore, the Zymogen granule membrane protein 16, a secretory lectin found in mucus which binds to and causes the aggregation of Gram-positive bacteria in the mouse gut thus retarding bacterial migration through mucus and into tissue [78], was significantly elevated by 14 dpi. Indeed an increase in mucus secretion is a characteristic feature of AGD, and although the fold changes were modest, the increase in the expression of several mucin protein isoforms observed in this study may pre-empt much larger expression increases at later stages of infection as suggested by results obtained from a genomic study showing up-regulation of mucin gene expression at 14 and 21 dpi [18].

In addition to the myriad immune factors which demonstrated differential expression in response to *P. perurans* colonisation, in the gill some proteins with roles in wound healing also exhibited differential expression upon disease progression. Polypyrimidine tract binding proteins have long been associated with cancer metastases [79], but have also been linked to the establishment of focal adhesions, a process which has been shown to be key in the dynamics of wound healing [80]. The calpains which belong to a ubiquitously expressed family of calcium-dependent cysteine proteases have been implicated in numerous cellular processes including apoptosis, bacterial clearance and wound healing [81,82,83]. Furthermore, members of the aminoacyl tRNA synthase complex-interacting multifunctional protein family have been shown to be secreted from macrophages at the site of tissue injury in response to TNFα, and have been implicated in wound healing by stimulation of fibroblast proliferation and collagen synthesis [84]. Taken together, the expression profiles of these proteins demonstrate an enhanced wound response only at the latest disease stage in this study (14 dpi), the point at which the most pronounced and numerous gross lesions are in evidence.

In the serum almost half of those proteins exhibiting significant differential expression across the various time points were found to have immune-related functions. Most, as discussed, were members of the apolipoprotein family, or the complement cascade. In addition thymosin beta a potent bacteriostatic agent which has been shown to be involved in the antimicrobial immune response in teleosts [85] and may also have a role in the acceleration of wound healing [86] was consistently up-regulated in the serum at the later timepoints. Glutathione peroxidase, a cellular anti-oxidant which also regulates the anti-viral immune response [87] was found here to be up-regulated in the serum but not the gill, which supports the findings of a previous study which demonstrated enhanced expression of peroxidases in serum but not mucus of AGD-affected salmon [18].

Hemagglutinin/amebocyte aggregation factor which has an established role in the mechanism of self/non-self-recognition [88] was down-regulated in serum. Transferrin is a protein associated with the innate immune response, which sequesters iron thus creating a low iron environment which is not conducive to bacterial survival [89]. Aside from a peak at 9 dpi, expression of the transferrins remained low or expression was decreased. Furthermore an increased expression of Olfactomedin 4 as observed in the serum has been linked to immune suppression and bacterial load in mice studies, and has been shown to suppress inflammation and immune cell infiltration [90].

In the serum an early immune response was observable. While further complement factor expression was detectable at the later time points, suppression of pro-immune factors and stimulation of immune-inhibitory factors became more apparent from 9 dpi. Conversely in the gill the traditional immune factors associated with classic early stage response showed little or no reaction to gill colonisation by *P. perurans*, while some immune specific inhibitory factors appeared stimulated, and other peripheral pathways involved in signalling and cell migration were depressed upon infection. Comparisons have previously been made between parasite induced host immunomodulation observed in helminthic parasite infection and immune gene expression in AGD [13]. Mechanisms of parasite immunosuppression appear to be varied and the impact profound [91]. The wide variety of factors negatively impacting on the immune response in this study support the concept of a pathogen-derived suppression of host response, as suggested in previous genomic studies [92].

## 5. Conclusions

Investigation of AGD in salmon gill and serum, at a proteomic level, across various sub-clinical and clinical time points has provided a novel insight into protein expression in very early disease stages. The results obtained demonstrated a lack of a robust classical immune response before 7 dpi in the gill, and many of the differentially expressed proteins identified have suppressive roles in the immune response and were not previously highlighted in the context of AGD. Conversely, some of the proteins differentially expressed in serum initially exhibited a positive immune response with some immune suppression evident at later stages. Taken together, the pro-immune trend observed in serum and the diversity of down-regulated proteins in the gill may indicate a local pathogen-derived suppression of host immune response. This study has identified many proteins which may have significant roles in the host response to AGD and may contribute to our understanding of the underlying pathology of the disease at both the local and systemic levels in particular during sub-clinical stages.

## Figures and Tables

**Table 1:** Immune related proteins differentially expressed in gills of AGD-affected salmon. Proteins were identified by mass spectrometry and analysis was performed by Max Quant and Perseus computational platforms. Values reported are *t*-test difference values (*p* value < 0.05; Q value < 0.001). Proteins present both in the gill and serum are highlighted in bold text.

Name	Gene	2 dpi	3 dpi	4 dpi	7 dpi	9 dpi	11 dpi	14 dpi	Peptide Matches	% Sequence Coverage
1-phosphatidylinositol 4,5-bisphosphate phosphodiesterase delta-1	A0A1S3QBL3				−1.76	−1.61		−1.35	6	22.00
Aminoacyl tRNA synthase complex-interacting multifunctional protein 2-like	A0A1S3S257				−1.82				2	26.6
**Apolipoprotein A-I isoform X1**	**A0A1S3NQ06**			**−0.91**					**33**	**78.2**
**Apolipoprotein A-I precursor**	**B5XBH3**			**−1.33**					**31**	**78.6**
**Apolipoprotein A-I-like**	**A0A1S3N6U9**				**2.08**			**2.72**	**7**	**34.50**
**Apolipoprotein A-IV**	**B5XCQ3**				**1.73**	**1.73**	**2.19**		**7**	**36.10**
**Apolipoprotein A-IV**	**B5X8U6**				**1.45**	**1.30**			**6**	**29.00**
**Apolipoprotein C-I isoform X1**	**A0A1S3RYY8**						**−0.62**		**8**	**55.2**
Apolipoprotein C-I-like	A0A1S3N6L4						−1.51		8	60.90
**Apolipoprotein Eb-like**	**A0A1S3RXT3**						**−2.40**		**17**	**59.80**
Apolipoprotein Eb-like	A0A1S3N6Y0						−0.83		18	69.1
Calcium uniporter protein, mitochondrial	A0A1S3QKG0						1.82		3	11.50
Calpain-2 catalytic subunit-like	A0A1S3Q7N2							−2.05	22	43.80
Calpain-9-like isoform X1	A0A1S3L6Q3							−1.85	17	32.50
Cation-dependent mannose—6-phosphate receptor	B5X109			2.01					3	10.40
**Complement C4-like(LOC106605920)**	**A0A1S3RYT8**				**−1.68**		**1.75**	**1.75**	**22**	**18.20**
Complement component C6 precursor	C0H9G0		0.45	1.16		0.63	0.59	0.71	6	7.8
**Complement factor H-like**	**A0A1S3QR20**							**1.28**	**37**	**50.1**
**Complement factor H-like**	**A0A1S3KK78**							**0.93**	**12**	**36.9**
**Complement factor I(cfi)**	**A0A1S3QGX2**	**1.92**	**1.89**	**1.68**		**1.94**	**1.92**	**1.37**	**6**	**13.10**
Deoxynucleoside triphosphate triphosphohydrolase SAMHD1-like(LOC106593439)	A0A1S3QJS3	−0.62		−1.59			−1.39		5	37.80
Dicarbonyl and L-xylulose reductase(dcxr)	B5X5V8				−2.10	−2.22	−2.96		3	13.10
DnaJ homolog subfamily A member 2	C0HBK7				6.13	4.73			11	24.80
DnaJ homolog subfamily C member 3-like	A0A1S3SDE5	−1.96							8	15.30
Eosinophil peroxidase-like(LOC106572735)	A0A1S3MI56			−2.11	−1.74	−1.47	−1.15	−2.05	34	50.50
Granulins-like isoform X2	A0A1S3NEN1		−2.12	−2.40			−2.09	−1.77	2	3.10
Heterogeneous nuclear ribonucleoprotein A0	B5X0T7						−2.12	−1.52	9	28.70
Histone H2A	B5X851							−1.80	7	41.40
Inositol-1-monophosphatase	N0GT48							−1.66	3	14.60
Intestinal mucin-like protein(LOC106595068)	106595068				1.05		0.78	1.29	26	39.10
Leucine-rich repeat-containing protein 59-like	A0A1S3Q6W9						−2.09		4	13.30
Leukocyte elastase inhibitor	B5X4J0		−1.06	−1.06	−1.59		−1.62		8	28.50
Magnesium transporter protein 1-like	A0A1S3QGC3				−2.23	−2.42			5	17.10
Mucin-2-like	A0A1S3QHF9				0.93			1.10	19	22.30
Mucin-5AC-like	A0A1S3R574				0.63				17	54.80
Mucin-5B-like	A0A1S3QG78						0.81		36	49.60
Myosin, light polypeptide 3-3	B5DGT3							−2.45	5	34.80
Nuclear Receptor Coactivator 5	B5X1C7					−2.71			10	16.00
Phosphatidylinositol 5-phosphate 4-kinase type-2 alpha isoform X3	A0A1S3NAV0			0.30				−1.53	4	11.40
Pollen-specific leucine-rich repeat extensin-like protein 1	A0A1S3QST0				1.73				4	23.40
Polypyrimidine tract-binding protein 2	B5X232							1.54	13	34.20
Polypyrimidine tract-binding protein 3-like	A0A1S3QGP4						−1.58	1.02	8	23.30
Ras-related protein Rab-11A(rb11a)	B5X4W2				−3.09	−3.01	−3.51	−2.73	9	39.40
Sodium/potassium-transporting ATPase subunit alpha-1-like(LOC106610479)	A0A1S3SLV8		−1.26	−0.96	−2.63	−2.81	−3.32		39	38.60
Trafficking protein particle complex subunit	C0H7U8				−1.76				4	24.40
Transgelin	B9ENC8							−1.52	19	77.70
Zymogen granule membrane protein 16-like	A0A1S3KWW8				0.81	0.94		1.84	5	38.50

**Table 2:** Immune related proteins differentially expressed in serum of AGD-affected salmon. Proteins were identified by mass spectrometry and analysis was performed by Max Quant and Perseus computational platforms. Values reported are ±*t*-test differences (*p* value < 0.05; Q value < 0.001). Proteins present in both gill and serum are highlighted in bold text.

Name	Gene	2 dpi	3 dpi	4 dpi	7 dpi	9 dpi	11 dpi	14 dpi	Peptide Matches	% Sequence Coverage
**Apolipoprotein A-I isoform X1**	**A0A1S3NQ06**						**−0.32**	**−0.31**	**48**	**85.5**
**Apolipoprotein A-I precursor**	**B5XBH3**			**−0.92**					**32**	**84.7**
**Apolipoprotein A-I-like**	**A0A1S3N6U9**						**1.65**	**1.66**	**27**	**74.1**
**Apolipoprotein A-IV**	**B5XCQ3**	**3.76**		**4.63**	**6.67**	**4.72**	**6.43**	**6.13**	**11**	**38.4**
**Apolipoprotein A-IV**	**B5X8U6**				**2.06**	**1.76**	**1.52**	**1.73**	**17**	**62.7**
Apolipoprotein A-IV-like	A0A1S3N6W3			1.49	1.21	1.58	1.42	1.36	21	61.6
**Apolipoprotein C-I isoform X1**	**A0A1S3RYY8**				**−1.29**				**9**	**60.9**
**Apolipoprotein Eb-like**	**A0A1S3RXT3**				**−0.99**		**−1.60**	**−1.06**	**21**	**54.1**
Apolipoprotein Eb-like	A0A1S3M355				1.91		2.87	2.77	11	35.2
CD59 glycoprotein-like	A0A1S3QTX5				1.07		1.01	1.28	4	48.9
Clusterin	C0H9Y2				0.70				8	15.8
Complement C1q subcomponent subunit C	C0HB93			2.30					4	14.5
Complement C1q-like protein 2	A0A1S3LRM2							0.85	7	28.1
Complement C1q-like protein 4 precursor	B9EPU5		0.82						10	42.9
Complement C1r-A subcomponent-like	A0A1S3RZ46		1.48			0.90			7	16.1
Complement C1r-A subcomponent-like isoform X2	A0A1S3MRC6	0.93							14	22.5
Complement C2-like	A0A1S3NRD5		0.43				0.75	0.80	41	52.6
Complement C3-like	A0A1S3L7F1			0.34	0.39		0.25		67	30.4
Complement C3-like	A0A1S3ML57					1.00			9	33.3
Complement C4-B	A0A1S3ML18					0.87	1.65	1.84	13	49
Complement C4-B-like	A0A1S3NRS7	1.01		1.13			0.80	0.84	28	13.6
Complement C4-like	A0A1S3T028	1.26		0.83					23	12.4
**Complement C4-like**	**A0A1S3RYT8**			**0.80**		**0.78**	**1.58**	**1.53**	**54**	**32.6**
Complement C4-like	A0A1S3QML7						1.82	1.83	35	50.1
Complement component C9	A0A1S3LT49	1.24		0.73	0.67		0.51	0.50	23	45.3
Complement factor B-like	A0A1S3LZP4	1.26	0.37	1.10	0.63		0.63		15	22.4
Complement factor B-like	A0A1S3L2E5		0.75	0.93		0.59	0.63	0.85	33	35.7
**Complement factor H-like**	**A0A1S3KK78**		**0.39**				**0.31**	**0.40**	**20**	**71.1**
**Complement factor H-like**	**A0A1S3QR20**			**0.40**	**0.42**				**48**	**50.3**
Complement factor H-like	A0A1S3KK91				0.87		1.37	1.42	6	32.4
**Complement factor I**	**A0A1S3QGX2**		**0.59**	**0.93**	**0.75**	**0.64**	**0.85**	**0.72**	**26**	**44.1**
Complement factor I-like	A0A1S3QGL8						0.61		13	47.8
Glutathione peroxidase	A0A1S3MM95						2.78	1.72	6	38.2
Hemagglutinin/amebocyte aggregation factor-like isoform X1	A0A1S3MZH7					−1.06	−1.07	−1.01	6	38.1
Olfactomedin-4-like	A0A1S3MMF4				0.96		1.21	0.88	7	12.4
Olfactomedin-4-like	A0A1S3KM81				0.93		1.20	0.99	9	18.3
Pentaxin	B5X672		−0.51			−0.65	−0.69	−0.77	13	55
Plasma protease C1 inhibitor-like	A0A1S3RGH0						0.53	0.47	25	39.8
Plasma protease C1 inhibitor-like	A0A1S3SJI8				0.59		0.81	0.79	14	26.2
Properdin-like	A0A1S3LII4					0.42	0.31	0.39	13	39.8
Serotransferrin	A0A1S2WYW0					1.50			82	78
Serotransferrin	A0A1S3R1S1						−0.35	−0.27	84	78.2
Serotransferrin-1-like	A0A1S3R1U4	0.62					−0.92	−0.85	25	43
Thymosin beta	B5XAM0		2.53			3.00	2.79	2.46	4	76.7

## Data Availability

Data are available via ProteomeXchange with identifier PXD022101.

## Supplementary Materials

The following are available online at https://www.mdpi.com/article/10.3390/microorganisms9040746/s1, Figure S1: Immune system process pathway analysis of serum samples using the Cluego app (v2.5.7) in Cytoscape (v1.5.7) demonstrated upregulation of pro-immune pathways at 3, 4 and 7 dpi (A–C). Immune enrichment was absent at 9 dpi, but was restored by 11 dpi (D). At 11 dpi and 14 dpi, the pro-immune pathway upregulation was accompanied by an elevation of various pathways which negatively regulate immunity (D,E). A *p* value of <0.05 and kappa coefficient of 0.4 were considered as threshold values. The size of the nodes reflects the statistical significance of the terms. No immune pathway enrichment was detected at any timepoint in gill samples.
