# Supplementary material for: Investigation of the Initial Host Response of Naïve Atlantic Salmon (Salmo salar) Inoculated with Paramoeba perurans
Source: Microorganisms. 2021 Apr 2;9(4):746. doi: 10.3390/microorganisms9040746 (PMC8066739; doi:10.3390/microorganisms9040746)
Supplement: Supplementary file 1 [file microorganisms-09-00746-s001.pdf]

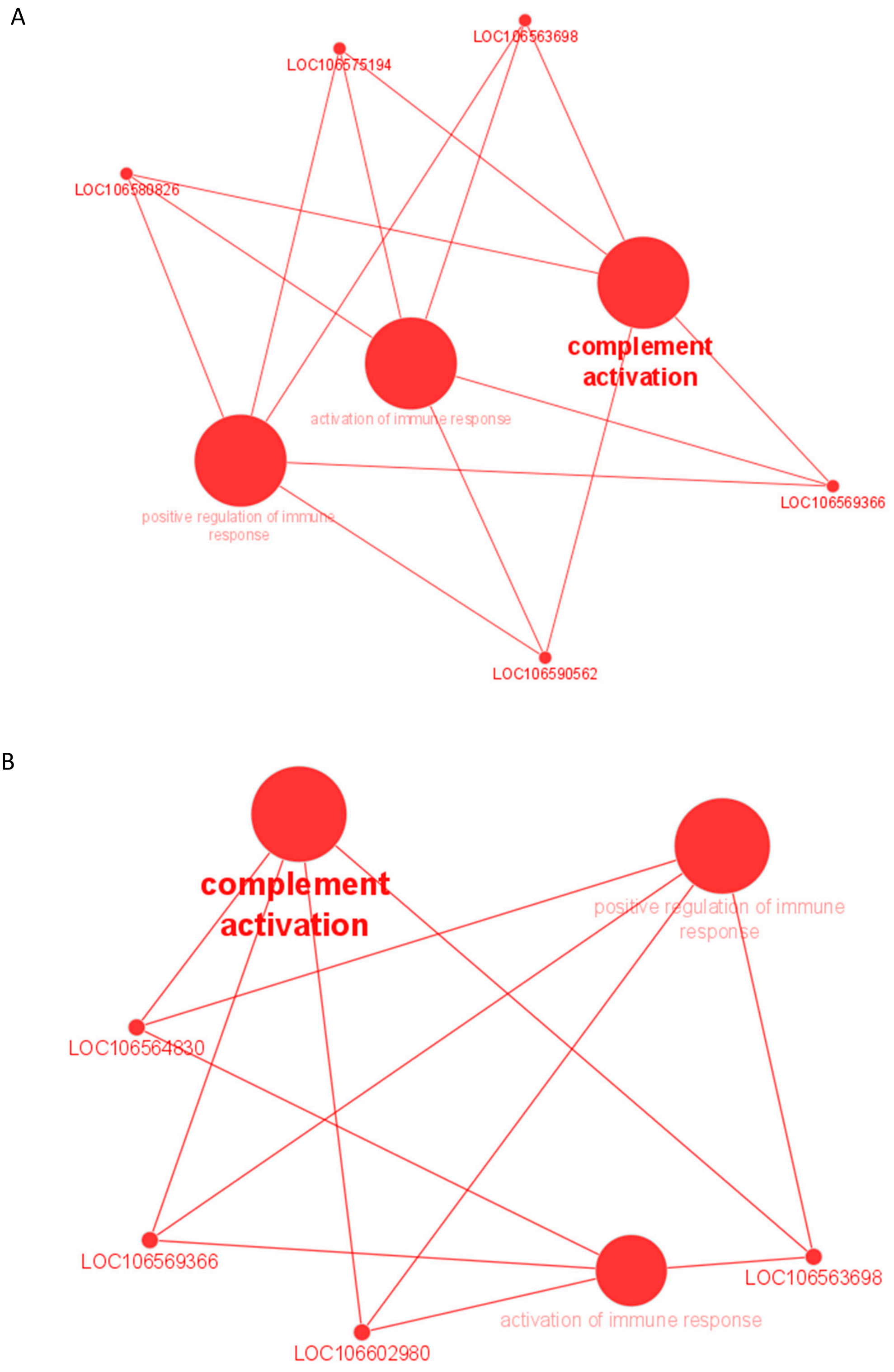
A
LOC106563698
LOC106575194
LOC106580826
complement activation
activation of immune response
positive regulation of immune response
LOC106569366
LOC106590562
B
complement activation
positive regulation of immune response
LOC106564830
LOC106569366
LOC106563698
activation of immune response
LOC106602980

C

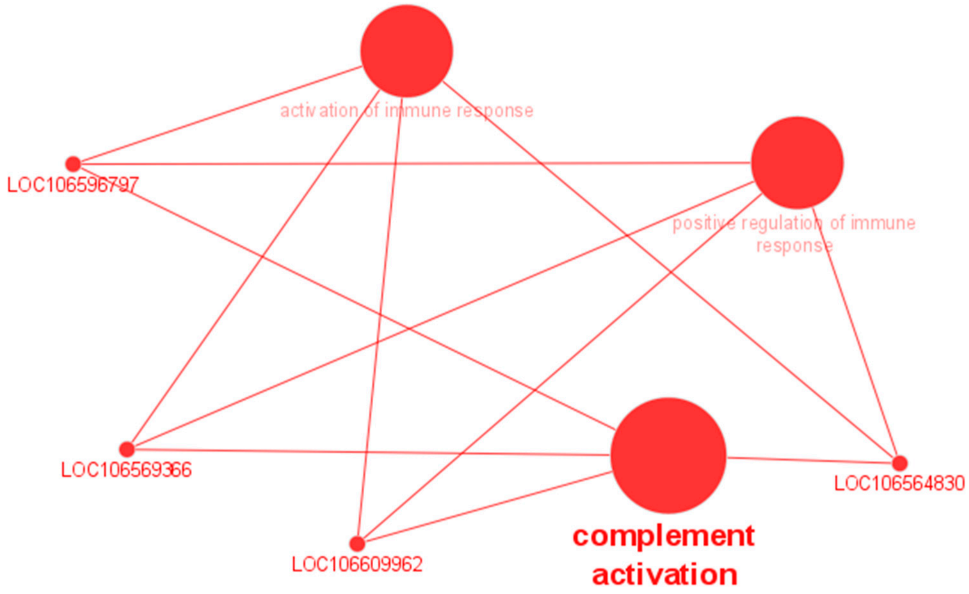
activation of immune response
LOC106596797
positive regulation of immune response
LOC106569366
LOC106564830
LOC106609962
complement activation

D

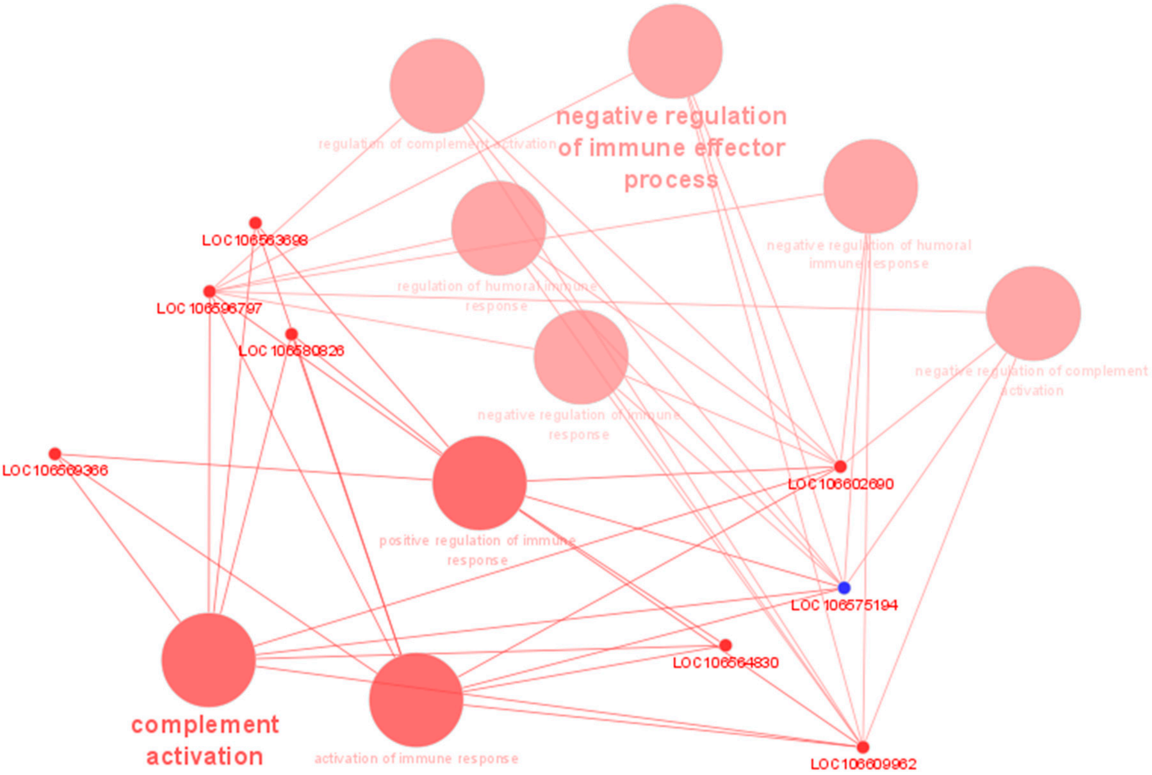
regulation of complement activation
negative regulation of immune effector process
negative regulation of humoral immune response
LOC106563698
regulation of humoral immune response
LOC106596797
LOC106580826
negative regulation of complement activation
negative regulation of immune response
LOC106569366
LOC106602690
positive regulation of immune response
LOC106575194
LOC106564830
complement activation
activation of immune response
LOC106609962

E

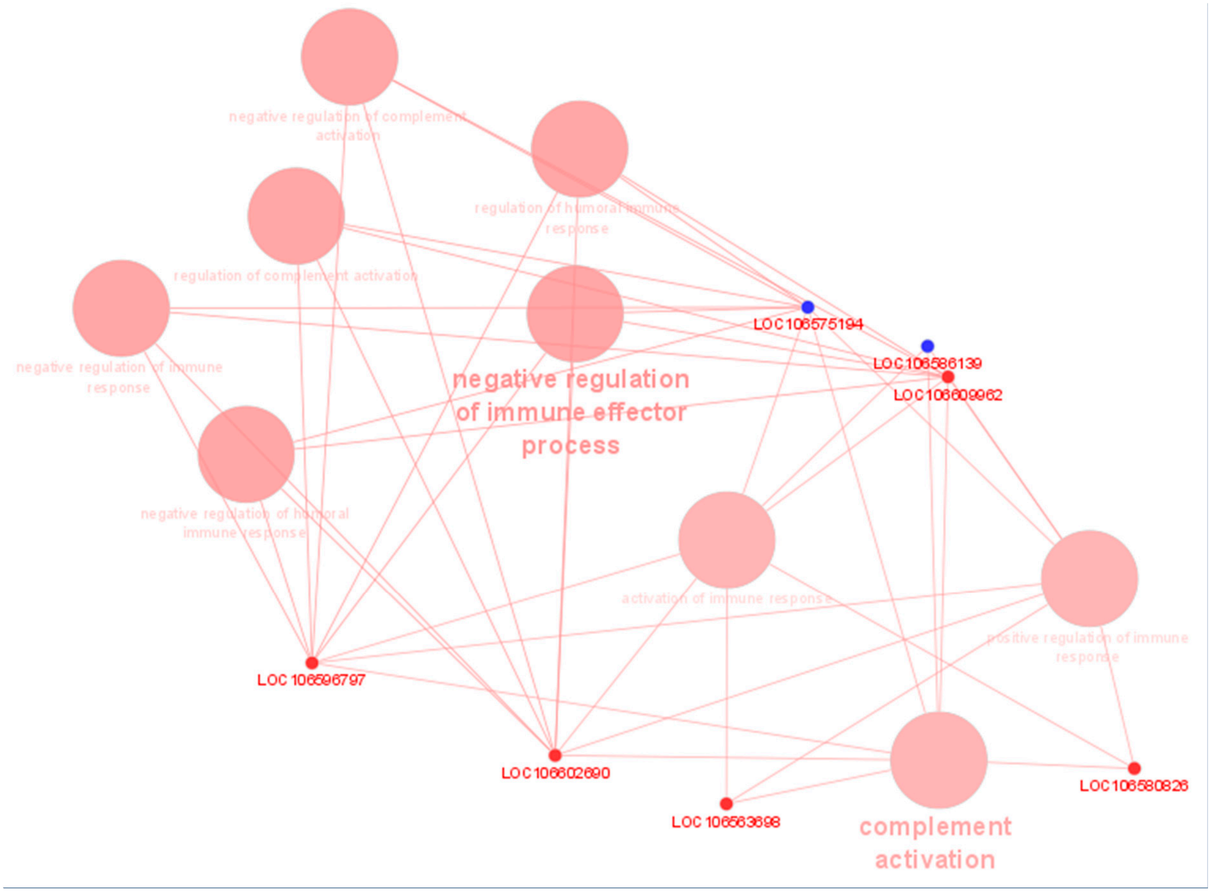

Figure S1. Immune system process pathway analysis of serum samples using the Cluego app (v2.5.7) in Cytoscape (v1.5.7) demonstrated upregulation of pro-immune pathways at 3, 4 and 7 dpi (A-C). Immune enrichment was absent at 9 dpi, but was restored by 11dpi (D). At 11dpi and 14 dpi, the pro-immune pathway upregulation was accompanied by an elevation of various pathways which negatively regulate immunity (D, E). A *P* value of <0.05 and kappa coefficient of 0.4 were considered as threshold values. The size of the nodes reflects the statistical significance of the terms. No immune pathway enrichment was detected at any timepoint in gill samples.
